# Notch Pathway Activation Contributes to Inhibition of C2C12 Myoblast Differentiation by Ethanol

**DOI:** 10.1371/journal.pone.0071632

**Published:** 2013-08-20

**Authors:** Michelle A. Arya, Albert K. Tai, Eric C. Wooten, Christopher D. Parkin, Elena Kudryavtseva, Gordon S. Huggins

**Affiliations:** 1 Molecular Cardiology Research Institute Center for Translational Genomics, Tufts Medical Center, Boston, Massachusetts, United States of America; 2 Genetics Program, Tufts University Sackler School of Biomedical Science, Boston, Massachusetts, United States of America; 3 Study Center on the Immunogenetics of Infectious Disease, Tufts University, Boston, Massachusetts, United States of America; University of Minnesota Medical School, United States of America

## Abstract

The loss of muscle mass in alcoholic myopathy may reflect alcohol inhibition of myogenic cell differentiation into myotubes. Here, using a high content imaging system we show that ethanol inhibits C2C12 myoblast differentiation by reducing myogenic fusion, creating smaller and less complex myotubes compared with controls. Ethanol administration during C2C12 differentiation reduced MyoD and myogenin expression, and microarray analysis identified ethanol activation of the Notch signaling pathway target genes Hes1 and Hey1. A reporter plasmid regulated by the Hes1 proximal promoter was activated by alcohol treatment in C2C12 cells. Treatment of differentiating C2C12 cells with a gamma secretase inhibitor (GSI) abrogated induction of Hes1. On a morphological level GSI treatment completely rescued myogenic fusion defects and partially restored other myotube parameters in response to alcohol. We conclude that alcohol inhibits C2C12 myoblast differentiation and the inhibition of myogenic fusion is mediated by Notch pathway activation.

## Introduction

Forty to sixty percent of chronic alcoholics suffer from weakness and locomotor difficulty [1], [2] caused by muscle damage [3] that is proportional to the duration and dose of their alcohol exposure [4]. Several mechanisms have been implicated in alcohol-induced myopathy including vitamin deficiency [5], [6], metabolic products of ethanol metabolism [7], and nutritional factors [8]. Because alcoholic myopathy is found in nutritionally intact individuals [1], alcohol is considered to have a direct myopathic effect. Alcohol has been found to affect muscle cells through multiple mechanisms including apoptosis [9], impaired protein synthesis [10] and kinase pathway activation [11]. Since alcohol can impair hepatocyte [12] and astrocyte [13] precursor cell differentiation we reasoned that alcohol may also contribute to muscle disease by inhibiting skeletal muscle repair through effects on the differentiation of myoblasts to myotubes.

The resident population of muscle stem cells, called satellite cells, repairs damaged muscle by differentiating into myoblasts that proliferate, differentiate, and fuse with existing myotubes [14]. Ethanol has been reported to inhibit rat primary skeletal myoblast proliferation and to delay differentiation [15]; however, the full effects of alcohol on the reparative myoblast are poorly understood. The C2C12 cell line was derived from activated murine satellite cells [16], and it is recognized as a cell model of myoblast to myotube differentiation that recapitulates transcription factor-regulated differentiation [16],[17]. An advantage of C2C12 cells in myoblast differentiation studies is the ability to synchronize the onset of differentiation with the conversion from growth medium to differentiation medium. Here, we sought to test our hypothesis that alcohol inhibits myoblast differentiation by studying its effects on C2C12 cell differentiation.

Transcriptional regulators that promote or inhibit myotube formation regulate skeletal myoblast differentiation. Positive regulators of myoblast differentiation include the basic helix-loop-helix (bHLH) muscle regulatory factors (MRF), MyoD and myogenin that are capable of activating the transcription of skeletal muscle genes [18], [19]. MyoD controls the commitment of precursor cells to the myoblast lineage, while myogenin regulates the differentiation of myoblasts to myotubes [18]. By comparison, the Notch signaling pathway inhibits myoblast differentiation [17] and promotes the less differentiated myoblast state [20] through multiple mechanisms, including expression of bHLH proteins that have transcriptional repression effects [17], [19]. The highly conserved canonical Notch signaling pathway is activated by an interaction between the Notch receptor and a ligand-bearing cell, causing gamma-secretase-mediated release of the Notch Intracellular Domain (NICD). NICD translocates to the nucleus where it interacts with the DNA binding protein CBF/RBP-Jk, thereby activating target gene transcription [17], [21]. The NICD-CBF transcriptional complex activates several target genes, including the hairy enhancer of split (Hes) family of transcriptional repressors, that negatively regulate tissue-specific transcription factors [22]. Expression of Hes1 and Hey1 transcripts identifies myoblasts activated by the Notch signaling pathway [23], [24].

In this study, we sought to define the cellular and transcriptional effects of alcohol on differentiating C2C12 myoblasts. We recognized that quantifying the effects of a small molecule on the size, shape and other properties of cells that stain positively for the myotube marker Troponin T (TnT) and counterstained for the nuclear stain DAPI is labor intensive, “low throughput”, and limited by investigator fatigue [25]. While anchorage-independent myoblast differentiation has been demonstrated [26], we did not favor automated flow assisted analysis of myotube properties because the asymmetric shape of myotubes is dependent on attachment to a tissue culture surface. Here, we demonstrate a method for systematic image recording of each well of a 96-well tissue culture dish coupled with computer-based image analysis capable of measuring the size and nuclear features of more than one thousand myotubes per well. Using this approach we demonstrate significant reductions in myotube size and complexity caused by alcohol treatment that are associated with both reduced MRF levels and increased expression of Notch pathway genes. We functionally confirm activation of the Notch signaling pathway by ethanol during C2C12 differentiation using a genetic reporter assay. Finally, we demonstrate that gamma-secretase inhibitor (GSI) treatment abrogates the effects of alcohol on myogenic fusion and modestly improves the morphology of myotubes which form from C2C12 myoblasts differentiating in the presence of ethanol.

## Materials and Methods

### Materials and Reagents

Ethanol was produced by PHARMCO-AAPER (Brookfield, CT). Rapamycin and L-685, 458 were purchased from Sigma (St. Louis, MO). Anti-mouse monoclonal Troponin T (TnT) antibody was obtained from the Hybridoma Bank (University of Iowa; Iowa City, IA). Taqman gene expression assays were purchased from Applied Biosystems (Foster City, CA). The pHes1-luc-tr firefly luciferase reporter plasmid contains a 350 bp fragment of the Hes1 promoter which is activated by NICD binding. The pCMV-Ren is a vector containing the renilla luciferase gene under a CMV promoter (Promega; Madison WI).

### Cell Culture, Transfection and Luciferase Assays

C2C12 mouse myoblasts (kindly provided by Dr Edgar Haber, Harvard University, Boston MA) were maintained at 37°C with 5% CO_2_ in growth medium (GM): DMEM-High Glucose medium containing 20% FBS and 1% penicillin-streptomycin (P/S) (Invitrogen; Grand Island, NY). Once cells reached 70% confluence, the medium was replaced with differentiation medium (DM): DMEM-High Glucose medium containing 2% horse serum and 1% P/S. The medium was changed daily during the course of differentiation. It was unnecessary to perform tests for mycoplasmal contamination. For plasmid transfection experiments C2C12 cells in GM were plated at a density of 10,000 cells/200 ul/well in 96-well plates. The following day the cells were transfected with 1 µg of DNA per well using Lipofectamine LTX and PLUS reagents (Promega; Madison, WI) and 24 h after transfection the medium was replaced with DM +/− ethanol. 24 h later the cells were harvested; firefly and renilla luciferase activity was measured. For each transfected well the firefly luciferase activity was first normalized to the renilla activity and treated cells were then normalized to control treated cells.

### C2C12 Differentiation Assay

C2C12 myoblasts differentiated in 96 well plates were fixed with 1% paraformaldehyde (Fisher; Pittsburgh, PA) (in PBS (Invitrogen)) and then permeabilized and blocked with 2% FBS (Invitrogen), 2% BSA (Fisher) and 0.2% Nonidet P40 (Fisher). Cells were probed with anti-TnT antibody (1∶200) followed by FITC-anti-mouse antibody (Invitrogen) (1∶1000) and then treated with DAPI (Fisher) in Glyercol (Fisher) and PBS. The stained cells were examined using the ImageXpress (Micro) (Molecular Devices Corporation), an inverted microscope imaging system to record thirty-six non-overlapping images from each well using a digital CCD camera at 10X magnification. Image analysis was performed using the MetaXpress Count Nuclei and Cell Scoring Application Module programs that was custom adapted to assess myoblast differentiation (method and program available upon request). The modified journal outlined the cell (region) based on FITC staining (TnT) and the region was overlayed onto images of the corresponding DAPI nuclear stain. DAPI staining nuclei that passed a pre-determined size cutoff and present within the outline of FITC staining were considered to represent myotube nuclei. The following myotube parameters were measured: area, perimeter, length, breadth, and number and area of myotube nuclei.

### Quantitative RT-PCR

Total RNA was extracted using the RNeasy Mini Kit (Qiagen; Valencia, CA), and cDNA was synthesized from 2 µg total RNA using random hexamers and the SuperScript III Reverse Transcriptase kit (Invitrogen). qPCR was performed on an ABI 7900 HT Fast Real Time PCR System using inventoried Taqman mouse primer/probes. Relative transcript abundances were determined by the standard curve method and normalized to Beta Actin and treated cells were normalized to control treated cells.

### Microarray Gene Expression Profiling

C2C12 cells seeded on 10 cm^2^ dishes in GM were treated with DM +/−100 mM ethanol until they reached 70% confluence. RNA was isolated using the RNeasy mini-kit (Qiagen), and RNA quality was assessed using the Agilent Bioanalyzer. The Yale Center for Genome Analysis performed transcript profiling on Illumina Mouse Ref8v2.0 microarrays (San Diego, CA). Raw expression values were processed by the Tufts Center for Neuroscience Research using Genome Studio. Basic quality control metrics were assessed as previously described [27]. Using R and the analysis package *lumi,* w*e* performed robust spline normalization (rsn), which is optimized to take advantage of the relatively high number of technical replicates randomly arrayed within each Illumina array [27], [28]. All normalized genes reported as present were passed to further analysis. Probes that changed with ethanol ±1.22 fold relative to controls with a p-value of ≤0.05 were considered significant and used for further analysis with Ingenuity Pathway Analysis (IPA) software (Ingenuity Systems; Redwood City, CA); pathways were considered highly relevant with a p-value ≤0.05 determined by Fisher’s Exact test. GENE-E software (Broad Institute; Cambridge, MA) was used to construct a heatmap of common genes differentially expressed between control and ethanol on all days; both arrays and genes were clustered using Spearman’s rank correlation coefficient and the colors in the heatmap are presented using the relative function which converts the expression values to heat map colors using the mean and maximum values for each row. The array data are available through the Gene Expression Omnibus accession number GSE46492.

### Statistical Analysis

All results are expressed as the mean ± standard error of the mean (SEM). Significance was determined by t-test or one-way ANOVA with Tukey test for multiple comparisons or two-way ANOVA followed by correction with either Bonferroni or multiple testing (FDR = 0.05) using Prism 5.0 software (GraphPad Software; LaJolla, CA) and MS Excel. A p value ≤0.05 was considered significant and indicated in graphs.

## Results

### A High-content and High-throughput Analysis of C2C12 Myoblast Differentiation

**Figure 1:**
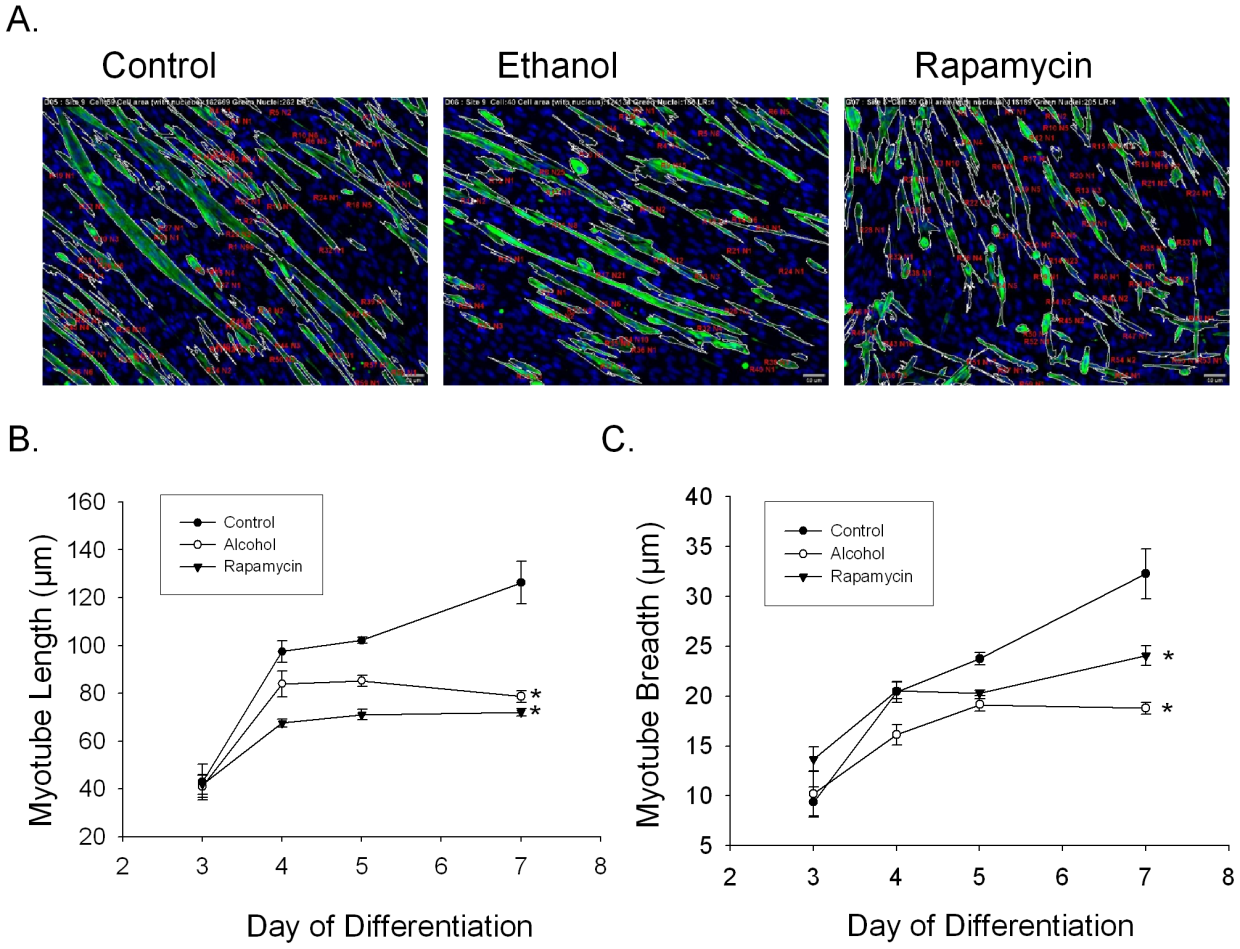
High Content Analysis of C2C12 differentiation. (A) Representative images of C2C12 cells differentiated for 7 days in control medium, medium containing ethanol (100 mM) or medium containing Rapamycin (100 nM). Myotubes are shown as identified and outlined by the image analysis system. (B) The mean ± SEM length (µm) and (C) breadth (µm) of C2C12 TnT staining myotubes between days 3 and 7 of differentiation. *p≤0.05 versus Control at day 7 (n = 3–15 independent replicates).

The ImageXpress (Micro) system was used to record 36 non-overlapping images from each well of a 96-well plate on channels that record FITC (myotubes staining for the TnT sarcomere protein) and DAPI (nuclei) signals. These studies produced 3,456 images per 96-well plate per channel. Digital superimposition of the FITC and the DAPI images allowed for computer-based identification of all TnT positive staining cells (myotubes) and the determination of which nuclei are present within a myotube (Figure 1A). Manual review of the images was performed to confirm accurate computer identification of myotubes (r^2^ = 0.9901, 95% CI: 0.9875–0.9980, p<0.001, m = 0.871). TnT-positive myotubes were initially found after 3 days of differentiation in DM; the number of myotubes increased significantly from days 3 to 7 (Figure S1A). A cellular analysis program was then used to measure myotube length, breadth, perimeter, area, nuclei number, and other features. In control treated C2C12 cultures myotube length, breadth, perimeter, area and nuclear number significantly increased from day 3 to day 7 of differentiation (Figure S1B–S1F). To evaluate whether the imaging system could identify the effects of a small molecule inhibitor of myoblast differentiation, we treated differentiating C2C12 cells with rapamycin, a known inhibitor of myoblast differentiation [29], [30]. Rapamycin treated C2C12 cells produced myotubes that had a significantly reduced length (Figure 1B), breadth (Figure 1C) and area, perimeter and fewer nuclei compared to myotubes that formed in control medium (Table 1). These studies demonstrate that our high-content imaging system capable of measuring thousands of myotubes from a 96-well plate can confirm the inhibitory effects of rapamycin.

**Table 1:** Comparison of C2C12 Myotube (TnT+) and non-myotube (TnT-) effects of alcohol.

Parameter	Vehicle Mean (SD)	Ethanol Mean (SD, *p*)	Rapamycin Mean (SD, *p*)
Length/Myotube (µm)	178 (11.3)	147 (13, *3.45E-14*)	117 (6.86, *1.35E-23*)
Breadth/Myotube (µm)	46.2 (5.3)	32.1 (2.33, *2.19E-12*)	30.0 (3.29, *2.95E-15*)
Area/Myotube (µm^2^)	4612 (982)	2405 (360, *3.35E-12*)	1586 (318, 1.93E-14)
Perimeter/Myotube (µm)	772 (111)	473 (59.7, *2.60E-15*)	405 (58.0, *3.69E-16*)
Nuclear area/TnT- Cell (µm^2^)	140 (7.37)	164 (8.81, *9.91E-16*)	169 (9.78, *1.78E-13*)
Cellular Integrated Intensity/Myotube (IIU)*	3550397 (555916)	2491590 (219876, *3.89E-10*)	1291084 (213302, 5.46E-18)
Myogenic Fusion Index (MFI)	14.9 (1.26)	13.5 (2.12, *6.99E-03*)	12.5 (2.03, *3.50E-05*)
Nuclei/Myotube	6 (1)	4 (1, *1.69E-13*)	3 (1, *2.16E-16*)
Myonuclear area (µm^2^)	133 (3.77)	140 (3.25, *1.99E-11*)	140 (3.42, 2.72E-08)

Mean (SD) from 24 independent experiments are shown.

### Ethanol Inhibits Myotube Formation

We next used our imaging system to determine whether alcohol affects C2C12 cell differentiation. Myotubes that formed over 7 days in differentiation medium supplemented with 100 mM ethanol, a high dose of alcohol that has been reported in humans [31] and found to affect calcium handling in cultured primary myoblasts [32], had significantly reduced length (Figure 1B) and breadth (Figure 1C) compared with control treated cells. After 7 days the alcohol treated cells were not significantly different from rapamycin treated cells.

Compared to control conditions, alcohol treated cells were significantly smaller as evidenced by a reduction in perimeter from 772 (SD = 111) µm in control medium to 473 (SD = 59.7) µm in ethanol, a reduction in length from 178 (SD = 11.3) µm to 147 (SD = 13) µm, a decrease in breadth from 46.2 (SD = 5.3) µm in control medium to 32.1 (SD = 2.33) µm with ethanol, and in area from 4612 (SD = 982) µm^2^ to 2405 (SD = 360) µm^2^ (Table 1). Further, C2C12 cells treated with 100 mM ethanol demonstrated a reduction in the number of nuclei per myotube from 6 (SD = 1) in control medium to 3 (SD = 1) in ethanol (Table 1). These results show that a high dose of ethanol impedes the differentiation of C2C12 myoblasts into myotubes.

Significant reductions in the Myogenic Fusion Index (MFI) were observed in alcohol treated cells compared with controls (Table 1) indicating the development of fewer fully differentiated myotubes. Further, the cellular integrated intensity, which is a measure of per myotube abundance of Troponin T, was also significantly reduced in alcohol treated cells compared with controls (Table 1). Collectively, alcohol treated cells were smaller with reduced myogenic fusion and Troponin T expression indicative of a defect in myoblast to myotube differentiation.

### Ethanol Inhibits MyoD and Myogenin Expression

**Figure 2:**
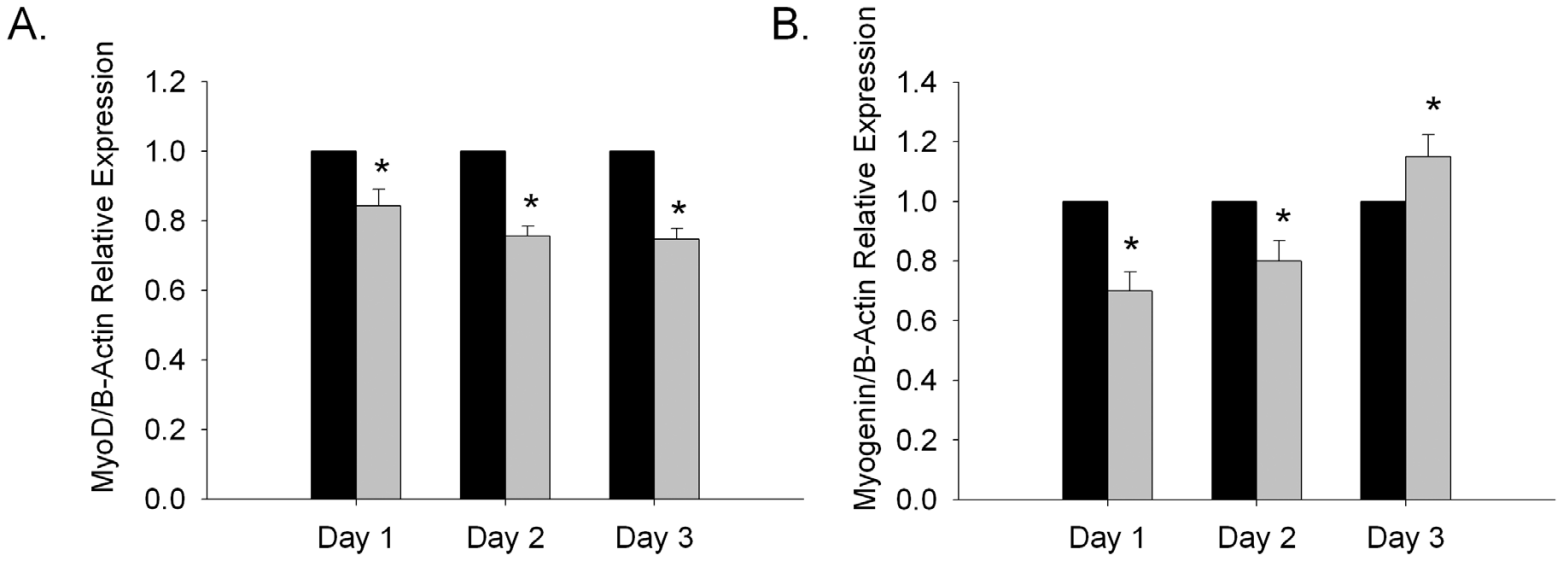
Ethanol down-regulates MyoD and Myogenin during C2C12 differentiation. C2C12 cells were induced to differentiate in DM +/−100 mM ethanol for 3 days. (A) MyoD and (B) myogenin mRNA levels were measured by qRT-PCR every 24 hours; transcript levels were quantified using the standard curve method and normalized to B-Actin. Black bars represent control medium treated cultures and grey bars represent cultures treated with medium containing alcohol. Mean ± SEM expression relative to non-alcohol control for each day are shown. *p≤0.05 versus control (n = 9 independent replicates).

Given our observation of smaller myotubes, we considered whether alcohol affected expression of the MRF transcription factors MyoD and myogenin that regulate C2C12 cell differentiation [33]. We wanted to only consider the levels of MRFs that contribute to myotube formation therefore we measured MRF levels using qRT-PCR upon induction of differentiation prior to when significant numbers of myotubes are visible. In control cells not treated with alcohol we observed an ∼50% decrease in MyoD expression and a robust increase in myogenin expression upon induction of differentiation (data not shown), as has been previously reported in C2C12 cells [33], [34]. Next we asked whether alcohol affected the expression of MyoD and myogenin by measuring their transcription during myoblast differentiation to myotubes. MyoD transcript levels were significantly lower in C2C12 cultures treated with DM supplemented with ethanol compared to control DM in each of three days of differentiation (Fig. 2A). Myogenin transcript levels were lower in ethanol treated cells compared with control DM for the first two days of differentiation (Fig. 2B) and increased slightly on day 3. These findings demonstrate that impaired myoblast to myotube differentiation by high dose ethanol correlates with reduced MyoD and myogenin transcript levels before the initial appearance of myotubes.

### Transcriptional Profiling Identifies Activation of the Notch Pathway by Ethanol during Myoblast Differentiation

**Figure 3:**
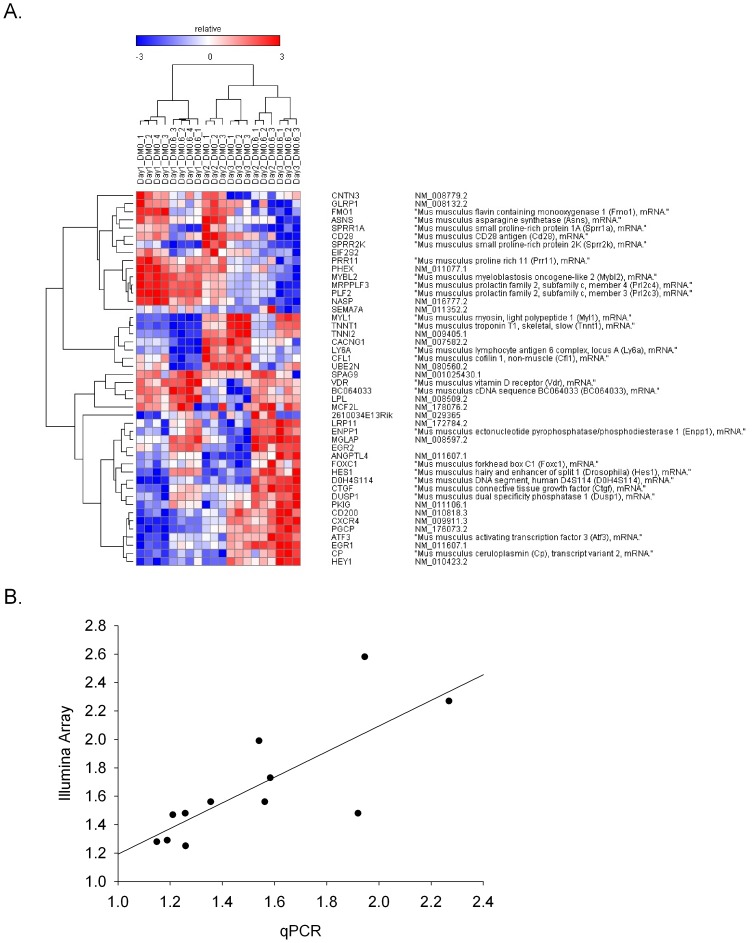
Expression profile of ethanol effects on differentiating C2C12 Cells. (A) RNA isolated from C2C12 cells induced to differentiate in control or ethanol (100 mM) containing DM for 1-day (n = 4 in each group), 2-days (n = 3 in each group) days and 3-days (n = 3 in each group) was hybridized to microarrays. Ingenuity pathway analysis was used to rank differentially expressed genes (fold-change = ±1.22). This analysis identified 46 genes that were differentially expressed between ethanol treated and control cells on all days of differentiation. Displayed genes are clustered using Spearman’s rank correlation coefficient. (B) Microarray log2 fold differences correlate with qRT-PCR log10 fold differences. Log ratios from the microarray were compared with qRT-PCR for 4 genes showing ≥1.22 fold-change (p≤0.05) on the microarray relative to control (Atf3, Hes1, Hey1 and Mgp). Correlation analysis yielded r = 0.6 (n = 3 independent replicates).

To determine whether alcohol affects additional pathways responsible for differentiation we performed gene expression profiling on RNA isolated from C2C12 cells on each of three days following the onset of differentiation in ethanol or control DM. We also analyzed the C2C12 cell transcription profile immediately prior to differentiation (time zero). IPA pathway analysis of the 3,583 transcripts significantly altered over the three-day period (Table S1) identified the Notch pathway as being significantly activated by ethanol (p = 0.004) (Table 2). In addition, the calcium signaling and the CXCR4 signaling pathways were significantly affected by ethanol during differentiation (p≤0.05). The relative expression levels of a subset of 46 common differentially expressed genes across all samples and days are represented in a heatmap (Fig. 3A). Hierarchical clustering accurately grouped the treatment groups together based upon transcriptional profile with the Day 3 profiles being most markedly different from Days 1 and 2. The Notch effectors Hes1 and Hey1 were among those genes that scored as differentially expressed. Additional Notch effectors, MyoR and BMP4 were significantly up-regulated on two of the three days of differentiation (1.29≤ fold-change ≤3.03; 1.75E-06≤p≤0.02). We found very good correlation of the ethanol-induced fold-differences in transcript level of microarray and by qPCR results for four genes (Atf3, Mgp, Hes1, Hey1; r = 0.6, Fig. 3B), validating the array findings. In summary, pathway analysis of gene expression profiling data identified activation of the Notch pathway associated with ethanol treatment during C2C12 differentiation.

**Table 2:** Canonical Pathways Activated by Alcohol Exposure.

Name	p-value	Ratio
Calcium Signaling	1.25E-03	4/207 (0.012)
Role of Tissue Factor in Cancer	2.98E-03	3/114 (0.026)
Notch Signaling	4.35E-03	2/43 (0.047)
Semaphorin Signaling in Neurons	8.02E-03	2/52 (0.038)
CXCR4 Signaling	8.37E-03	3/169 (0.018)

### Notch Target Genes are Activated by Ethanol during C2C12 Differentiation

**Figure 4:**
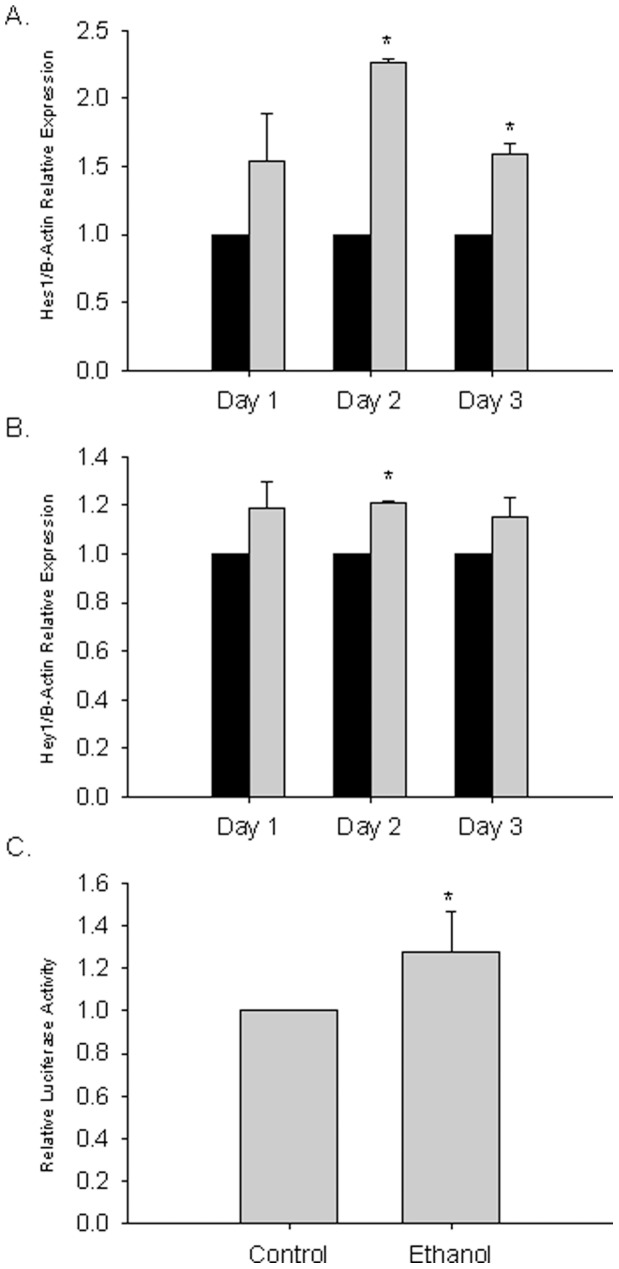
Ethanol activates the Notch signaling pathway. (A) Hes1 and (B) Hey1 mRNA levels measured by qRT-PCR in C2C12 cells induced to differentiate for 2 days in control (black bars) or DM containing 100 mM (shaded bars) ethanol. Mean ± SEM expression normalized to B-Actin relative to untreated controls is shown (asterisk indicates Student’s t-test p≤0.05 (n = 3 independent replicates). (C) C2C12 cells were transiently transfected with pHes1-luc-tr in GM followed by treatment for 1 day in control or DM containing 100 mM ethanol. The firefly luciferase activity was normalized to renilla luciferase for each sample, and the mean ± SEM relative luciferase activity relative to untreated controls from three independent experiments is shown (asterisk indicates Student’s t-test *p*≤0.05).

Analyzing independent samples we confirmed significant up-regulation of Hes1 in differentiating C2C12 cells by ethanol compared to control DM over three days of differentiation (Fig. 4A). The Hey1 transcript was increased on all days of differentiation (Fig. 4B). To further evaluate Notch-dependent effects of alcohol we tested whether alcohol activated a luciferase reporter plasmid regulated by the Hes1 proximal promoter containing the well-characterized CSL-Notch-ICD binding sites [35] in C2C12 cells. We found that ethanol significantly activated the proximal Hes1 promoter. (Fig. 4C). These results functionally confirm ethanol activation of the Notch pathway during C2C12 differentiation, with a magnitude that is similar to ethanol’s induction of transcription of Notch target genes, Hes1 and Hey1.

### GSI Treatment Blocks Alcohol Activation of Hes1 in C2C12 Cells

**Figure 5:**
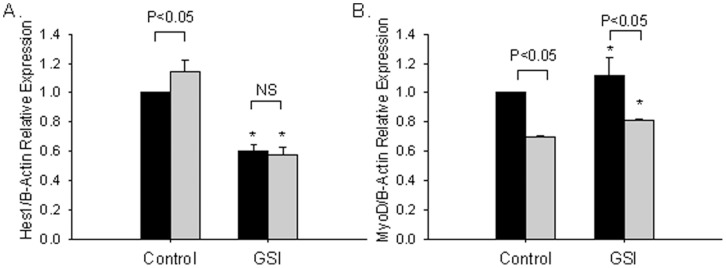
GSI treatment blocks alcohol-dependent Hes1 activation. C2C12 cells were induced to differentiate for 2 days in control, DM containing either 100 mM ethanol, 1 uM L-685, 458 or both. Mean ± SEM (A) Hes1 (n = 4 independent replicates) and (B) MyoD (n = 2 independent replicates) mRNA transcript levels measured by qRT-PCR relative to untreated controls is shown. Black bars represent control medium treated cultures and grey bars represent cultures treated with medium containing alcohol. GSI treatment significantly reduced Hes1 expression in both control cells and cells exposed to alcohol. Induction of Hes1 by alcohol administration in the absence of GSI treatment was not observed in the presence of L-685, 458 consistent with activation of the Notch pathway by alcohol. L-685, 458 treatment mildly increased MyoD expression with a partial reversal of alcohol mediated repression. Asterisk indicates Student’s t-test *p*≤0.05 and NS indicates non-significant.

To confirm that the increase in endogenous Hes1 expression by ethanol was mediated via the Notch pathway we differentiated C2C12 cells in the presence of ethanol and the GSI L-685,458. L-685, 458 prevents the final cleavage of the Notch receptor and therefore the release of the intracellular domain [36]. The effect of L-685, 458 on Hes-1 levels caused a decrease of ∼ 50% compared to controls (Fig. 5). This would be expected if low level endogenous Notch signaling is blocked. While alcohol significantly increased Hes1 expression, the addition of L-685,458 abolished this effect (Fig. 5A) to levels seen with L-685, 458 in control conditions which confirms that induction of Hes-1 by alcohol is mediated via activation of Notch signaling. Because overexpression of the Notch-ICD in C2C12 cells has been demonstrated to decrease MyoD expression [23] we next asked whether GSI treatment also blocked the observed effects of alcohol on MyoD expression. GSI treatment alone mildly increased levels of MyoD. Although MyoD levels increased in the presence of GSI and alcohol, MyoD levels remained significantly reduced in GSI and alcohol treated cells compared with GSI treatment alone (Fig. 5B). These findings are consistent with alcohol activating Notch-dependent transcription of Hes1 and reducing MyoD transcript levels partly through a Notch–dependent mechanism.

### GSI Treatment Improves C2C12 Differentiation and Partially Restores Ethanol-dependent Effects

**Figure 6:**
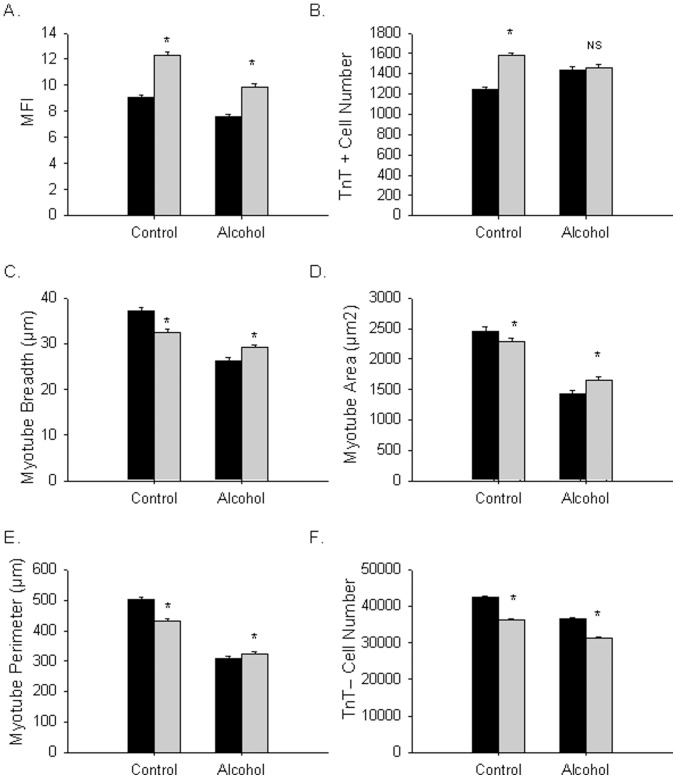
GSI treatment restores ethanol effects on myogenic fusion and partially restores morphology of C2C12 myotubes. C2C12 cells were induced to differentiate for 7 days in control, DM containing 100 mM ethanol, 1 uM L-685, 458 or both. Mean ± SEM values for (A) myogenic fusion index (MFI), (B) Number of myotubes, (C) breadth (µm), (D) area (µm^2^), (E) perimeter (µm), and (F) number of undifferentiated cells are shown. A statistical interaction was observed between ethanol and GSI for MFI, myotube number, myotube breadth, myotube perimeter and area two-way ANOVA (P<0.001 for interaction). The interaction was non-significant for number of undifferentiated cells. Black bars are cells without GSI treatment and shaded bars are cells with GSI treatment. Significant differences between control and ethanol were found both with and without GSI (asterisk indicates ANOVA *p*≤0.05 compared with non-alcohol treated control) (n = 24 independent replicates).

Finally we tested whether GSI treatment improved the cellular effects of alcohol on myoblast differentiation. C2C12 cells were treated with DM supplemented with vehicle, alcohol, the GSI L-685, 458, or alcohol and L-685, 458. Compared to vehicle alone, GSI treatment significantly increased the number of TnT+ cells and the MFI. Next, we analyzed the effect of GSI on alcohol-dependent effects by a two-way ANOVA to determine whether a statistically significant interaction could be found between the alcohol and GSI treatments. The presence of a significant interaction would support that a biological effect of alcohol is in part mediated by activation of the Notch pathway. We observed a significant interaction between alcohol and L-685, 458 for myogenic fusion index (MFI) with the GSI completely restoring this property (p<0.001, Fig. 6A). We also observed a significant interaction between alcohol and L-685, 458 for number of myotubes (p<0.0001, Fig. 6B), myotube breadth (p<0.0001, Fig. 6C), and area (p = 0.03, Fig. 6D) consistent with some of alcohol’s effects resulting from a Notch-dependent mechanism. The interaction between GSI and ethanol was non-significant for numbers of undifferentiated cells (p>0.05, Fig. 6E) suggesting that the pool of differentiation committed cells is increased in the dually treated cells due to blockage of endogenous Notch signaling by GSI and also by Notch-independent mechanisms. Since GSI treatment did not restore myotube parameters to control levels or to levels seen with GSI treatment alone this suggests that in addition to Notch dependent effects there are also significant Notch-independent effects of alcohol that are responsible for alcohol effects on myoblast differentiation.

## Discussion

We measured differences in the cellular and transcriptional profiles of C2C12 myoblasts undergoing differentiation in the presence of control or ethanol-containing medium. To provide greater power to detect changes in myoblast differentiation caused by ethanol, we developed a high-content immunocytochemistry screen. Our imaging approach, which captures over 50% of the surface of each well contained within a 96-well tissue culture plate followed by computer-aided myotube measurement, was designed to address the weaknesses of many immunocytochemistry screens of adherent myoblasts, principally, analysis of a small portion of the available cell culture surface and measurement of a limited number of cell phenotypes. Our high-content screen successfully identified previously observed [29], rapamycin-induced reductions in cellular size and nuclear content. Strikingly, our studies also demonstrate significant defects in myoblast differentiation caused by alcohol administered at the onset of differentiation. Gariga et al. previously demonstrated that alcohol reduces primary rat myoblast proliferation and delays differentiation when administered to undifferentiated cells [15]. We identify reductions in multiple myotube parameters, including reduced MFI and integrated intensity of Troponin T staining, without time-dependent improvements in the myotubes, consistent with a defect in differentiation. The differences in myotube parameters are preceded by significant reductions in MyoD and myogenin expression during the first 48-hours of differentiation, suggesting that rapid effects on transcriptional regulators of myoblast differentiation may be at least partly responsible for the abnormal myotube phenotypes caused by alcohol. Our study demonstrates that high dose alcohol may cause myopathy, at least in part, by damaging the ability of differentiating muscle precursor stem cells to repair damaged myocytes.

Ligand activation of the Notch pathway triggers release of the Notch-ICD that translocates to the nucleus where among many effects causes up-regulation of Hes1 [23]. Ethanol has also been shown to enhance CBF-1/RBP-Jk promoter activity in human umbilical vein endothelial cells [37] and to decrease activity in vascular smooth muscle cells [38]. Here, we demonstrate that alcohol activates Hes1 expression in C2C12 cells in a manner sensitive to a GSI, and alcohol also modestly activated the proximal Hes1 promoter regulated by CBF/RBP-Jk binding sites. While the transcriptional differences in Hes1 and Hey1 in the first 48-hours of differentiation were modest our finding that GSI treatment interacted statistically with alcohol treatment and restored myogenic fusion is consistent with alcohol modulating myoblast differentiation in part through the Notch pathway. Since significant myotube defects remained in alcohol treated cells in the presence of GSI treatment we acknowledge that alcohol does not inhibit myoblast differentiation solely through a Notch–dependent mechanism, rather our pathway analysis and our inhibitor studies are consistent with both Notch-dependent and –independent effects of alcohol.

Activation of the Notch pathway has been known to inhibit myoblast differentiation for over a decade [39], however, the precise mechanism of this effect remains uncertain. Hes1 expression is regulated by the Notch pathway. However, Hes1 expression is not always increased when the Notch pathway inhibits myoblast differentiation and Hes1 overexpression does not inhibit C2C12 cell differentiation [40]. Nofziger et al. demonstrated that ligand induced Notch activation can occur independently of CBF1 and is sufficient to block C2C12 myoblast differentiation [41]. By comparison, overexpression of Hey1 is sufficient to inhibit MyoD-induced muscle cell differentiation [42] and C2C12 cell differentiation; however, Hey1 is not necessary for the Notch pathway to inhibit myotube formation [17], suggesting that additional Notch regulated genes are involved. Indeed, Notch inhibits C2C12 cell differentiation through multiple pathways including the transcription factor MyoR [17], which our microarray analysis found to be up-regulated in C2C12 cells treated with ethanol. Our analysis also demonstrated up-regulation of BMP4, which has been demonstrated to inhibit C2C12 cell differentiation in a manner sensitive to GSI treatment or expression of a dominant negative version of CSL [36]. In addition to Notch, significant differences in the calcium signaling pathway were induced by ethanol treatment during C2C12 differentiation. Acute ethanol treatment alters calcium handling in primary myocytes [32], and alterations in calcium handling have been reported to inhibit myogenin and Myosin Heavy Chain gene expression and myotube formation [43]. Taken together our microarray study is consistent with ethanol causing activation of multiple Notch target genes as well as Notch-independent pathways that collectively inhibit C2C12 cell differentiation.

We acknowledge that the concentration of alcohol used in our studies is higher than generally recorded in alcoholics, however, even higher blood alcohol levels have been found in humans [44] and while we refreshed the culture medium daily our cell culture system inherently allowed for loss of alcohol through evaporation. We further acknowledge that small molecule inhibitors can have off-target effects, however the GSI used in this study has been described to be a specific and potent inhibitor of gamma secretase [45], suggesting that reductions of Hes1 expression and improvements in myogenic fusion index caused by L-685,458 treatment are consistent with effects on the Notch pathway. Taken together we show that inhibition of C2C12 myoblast differentiation by alcohol correlates with activation of the Notch signaling pathway and reduced MRF expression. Recently, clinical studies have identified well tolerated GSI compounds [46], raising consideration whether GSI treatment could be considered for clinical investigation of alcohol-induced myopathies. Our studies suggest that further investigation is warranted to assess whether GSI treatment has potential to partially improve muscle function in patients suffering from alcohol-associated muscle disease.

## Supporting Information

Figure S1 **C2C12 Cell Differentiation is Ongoing at 7 days.** C2C12 cells were differentiated as previously described. The mean ± SEM of (A) myotube number, (B) myotube nuclei, (C) myotube length, (D) myotube breadth, (E) myotube perimeter, (F) myotube area in TnT staining C2C12 myotubes between days 3 and 7 of differentiation. *p≤0.05 versus Day 3 (n = 3–15).
(PDF)

Table S1 (XLSX)
