# Supplementary figures and images for: Notch Pathway Activation Contributes to Inhibition of C2C12 Myoblast Differentiation by Ethanol
Source: PLoS One. 2013 Aug 20;8(8):e71632. doi: 10.1371/journal.pone.0071632 (PMC3748126; doi:10.1371/journal.pone.0071632)

## Supplemental Figure S1

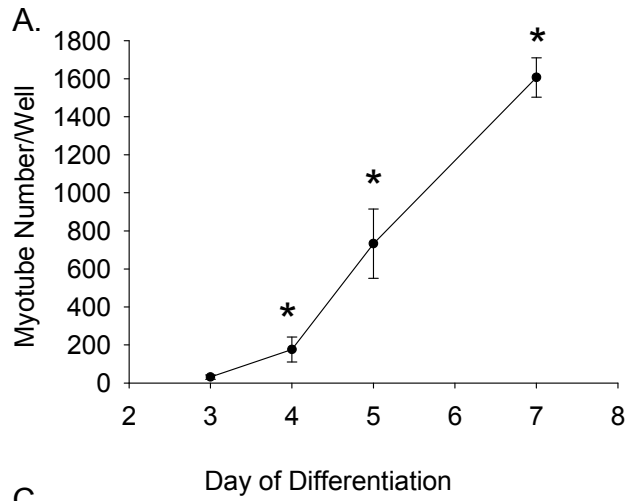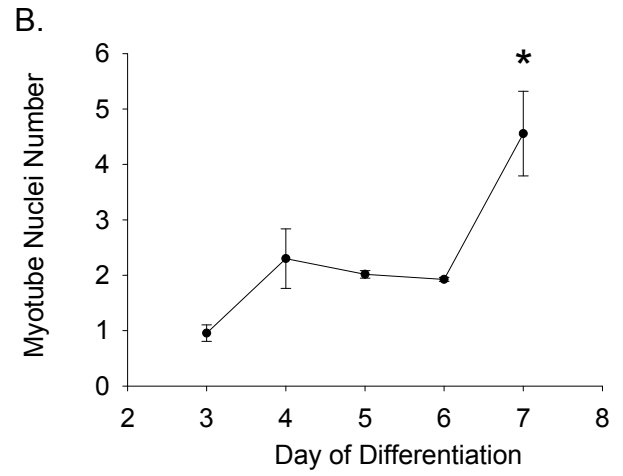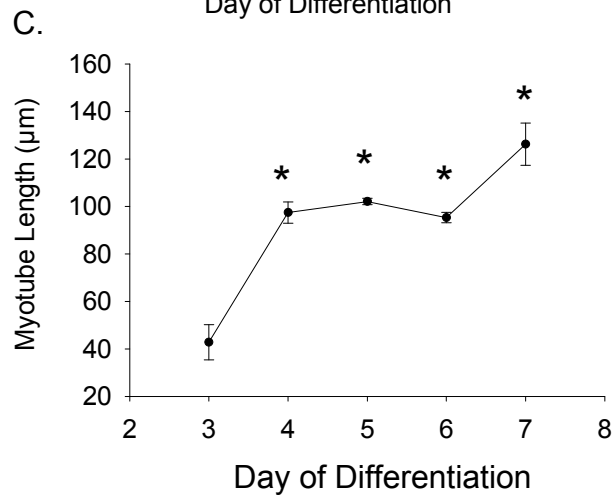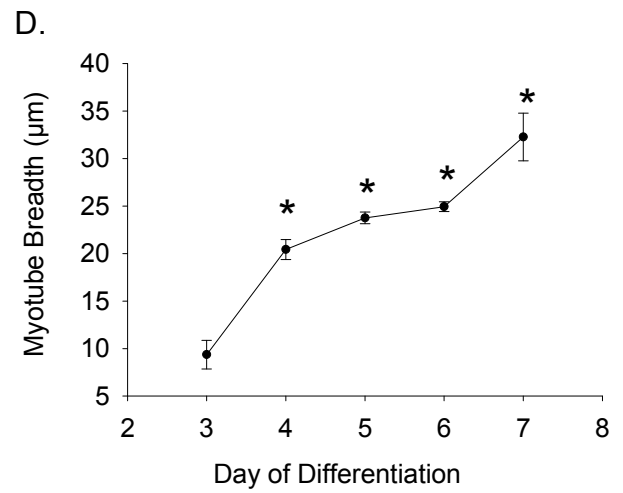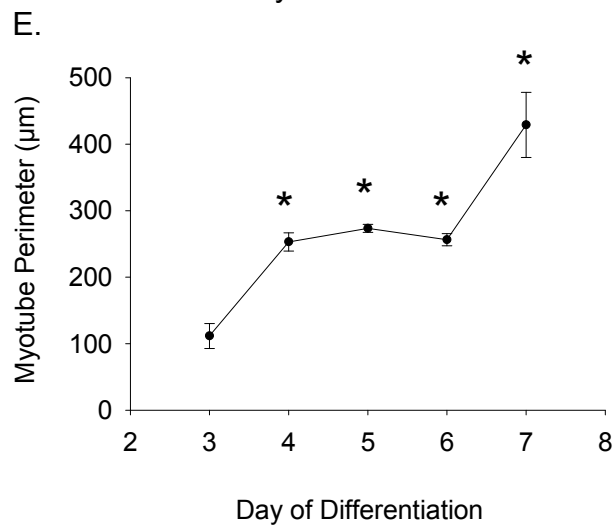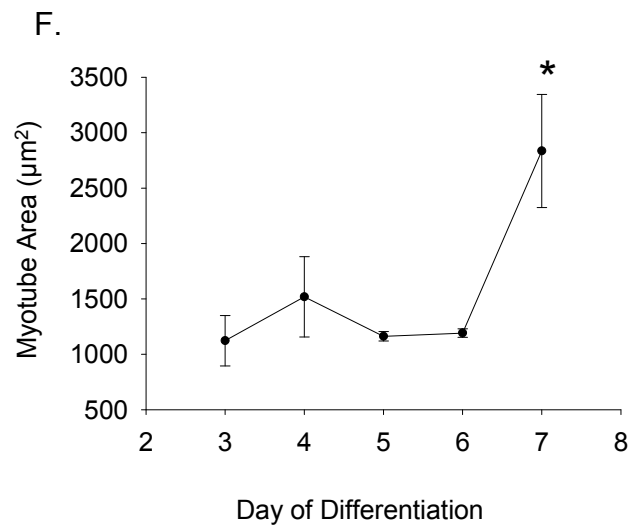

Supplement: Figure S1 — C2C12 Cell Differentiation is Ongoing at 7 days. C2C12 cells were differentiated as previously described. The mean ± SEM of (A) myotube number, (B) myotube nuclei, (C) myotube length, (D) myotube breadth, (E) myotube perimeter, (F) myotube area in TnT staining C2C12 myotubes between days 3 and 7 of differentiation. *p≤0.05 versus Day 3 (n = 3–15). (PDF) [file pone.0071632.s001.pdf]
